# Genetic causes of male infertility: snapshot on morphological abnormalities of the sperm flagellum

**DOI:** 10.1186/s12610-019-0083-9

**Published:** 2019-03-04

**Authors:** Jean-Fabrice Nsota Mbango, Charles Coutton, Christophe Arnoult, Pierre F. Ray, Aminata Touré

**Affiliations:** 1 INSERMU1016, CNRS UMR8104, Université Paris Descartes, 75014 Paris, France; 2 Centre National de la Recherche Scientifique UMR8104, 75014 Paris, France; 3 Faculté de Médecine, Université Paris Descartes, Sorbonne Paris Cité, 75014 Paris, France; 4 Institut for Advanced Biosciences, Université Grenoble Alpes, INSERM U1209, CNRS UMR 5309, 38000 Grenoble, France; 5 CHU Grenoble Alpes, UM de Génétique Chromosomique, Grenoble, France; 6 CHU de Grenoble, UM GI-DPI, F-38000 Grenoble, France

**Keywords:** Male infertility, Genetic, MMAF, Sperm, CFAP, WDR, DNAH, AK, Infertilité masculine, Génétique, MMAF, Spermatozoïde, CFAP, WDR, DNAH, AK

## Abstract

Male infertility due to Multiple Morphological Abnormalities of the sperm Flagella (MMAF), is characterized by nearly total asthenozoospermia due to the presence of a mosaic of sperm flagellar anomalies, which corresponds to short, angulated, absent flagella and flagella of irregular calibre. In the last four years, 7 novel genes whose mutations account for 45% of a cohort of 78 MMAF individuals were identified: *DNAH1*, *CFAP43*, *CFAP44*, *CFAP69*, *FSIP2*, *WDR66 (CFAP251), AK7*. This successful outcome results from the efficient combination of high-throughput sequencing technologies together with robust and complementary approaches for functional validation, in vitro, and in vivo using the mouse and unicellular model organisms such as the flagellated parasite *T. brucei*. Importantly, these genes are distinct from genes responsible for Primary Ciliary Dyskinesia (PCD), an autosomal recessive disease associated with both respiratory cilia and sperm flagellum defects, and their mutations therefore exclusively lead to male infertility. In the future, these genetic findings will definitely improve the diagnosis efficiency of male infertility and might provide genotype-phenotype correlations, which could be helpful for the prognosis of intracytoplasmic sperm injection (ICSI) performed with sperm from MMAF patients. In addition, functional study of these novel genes should improve our knowledge about the protein networks and molecular mechanisms involved in mammalian sperm flagellum structure and beating.

## Résumé

Les infertilités masculines dues au phénotype de « flagelles courts » ou « Multiple Morphological Abnormalities of the sperm Flagella » (MMAF), sont caractérisées par une asthénozoospermie quasi totale associée à la présence d’une mosaïque d’anomalies flagellaires correspondant à des flagelles courts, angulés, absent ou de calibre irrégulier. Durant les quatre dernières années, une approche génétique par séquençage d’exome de 78 patients MMAF a permis l’identification de mutations causales dans 7 gènes: *DNAH1*, *CFAP43*, *CFAP44*, *CFAP69*, *FSIP2*, *WDR66 (CFAP251), AK7,* permettant ainsi un diagnostic pour près de 45% des sujets de la cohorte. Ce succès remarquable résulte de la combinaison efficace de technologies de séquençage à haut débit et d’approches complémentaires de validation fonctionnelle des mutations, in vitro et in vivo, dans le modèle murin et les modèles unicellulaires tels que le parasite flagellé *T. brucei*.

De manière importante, les gènes identifiés sont distincts des gènes responsables de Dyskinésie Ciliaire Primitive (DCP), une maladie autosomale récessive associée à des défauts des cils et du flagelle, et leurs mutations induisent par conséquent une infertilité masculine isolée. Dans le futur, ces résultats génétiques vont permettre d’améliorer le diagnostic des infertilités masculines humaines et potentiellement de fournir des corrélations génotype-phénotype, utiles pour le pronostic de la fécondation in vitro par injection intra-cytoplasmique des spermatozoïdes de sujets MMAF. Par ailleurs, les études fonctionnelles de ces nouveaux gènes identifiés, permettront de mieux définir les mécanismes moléculaires et les complexes protéiques impliqués dans l’assemblage et le battement du flagelle.

## Introduction

**Fig. 1:**
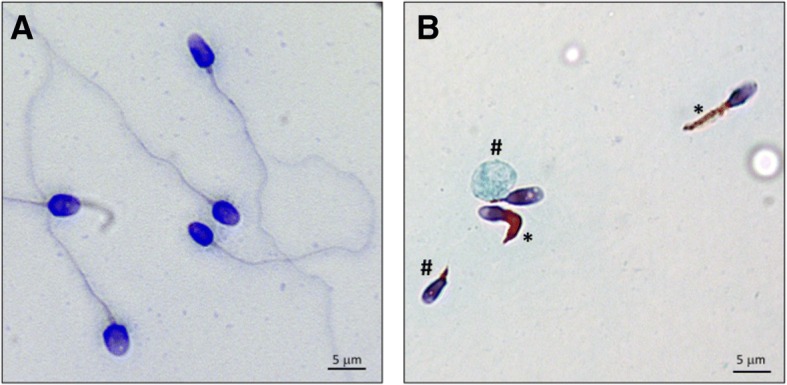
Morphological defects of the MMAF phenotype. (**a**) control individual; (**b**) MMAF individual. Picture from Aminata Touré. Analysis by photon microscopy shows the presence of a mosaic of morphological defects in semen from MMAF patients; in particular, sperm cells with absent (#) and short flagella (*)

In the last decade, tremendous work was performed in the field of reproductive biology in order to identify genetic causes of male infertility. Such work was greatly facilitated by the emergence of high throughput sequencing technologies, which allowed rapid identification of several genes required for sperm production and function [1, 2]. This mini-review focuses on male infertility due to Multiple Morphological Abnormalities of the sperm Flagella (MMAF), a phenotype previously identified as ‘dysplasia of the fibrous sheath’, ‘short tails’ or ‘stump tails’ [3–5]. MMAF is characterized by nearly total asthenozoospermia due to the presence of a mosaic of sperm flagellar anomalies which corresponds to short, angulated, absent flagella and flagella of irregular calibre (Fig. 1). The proportion of these anomalies is variable between MMAF patients but all are constantly present at levels largely exceeding those found in control men. Hence, recent study of a cohort of 78 MMAF patients indicated an average of 40% of spermatozoa with short flagella and 20% with no flagella [6] while only 1 and 5% of these anomalies, respectively, are recorded in semen from a control population of 926 fertile men (95th percentile) [7].

**Fig. 2:**
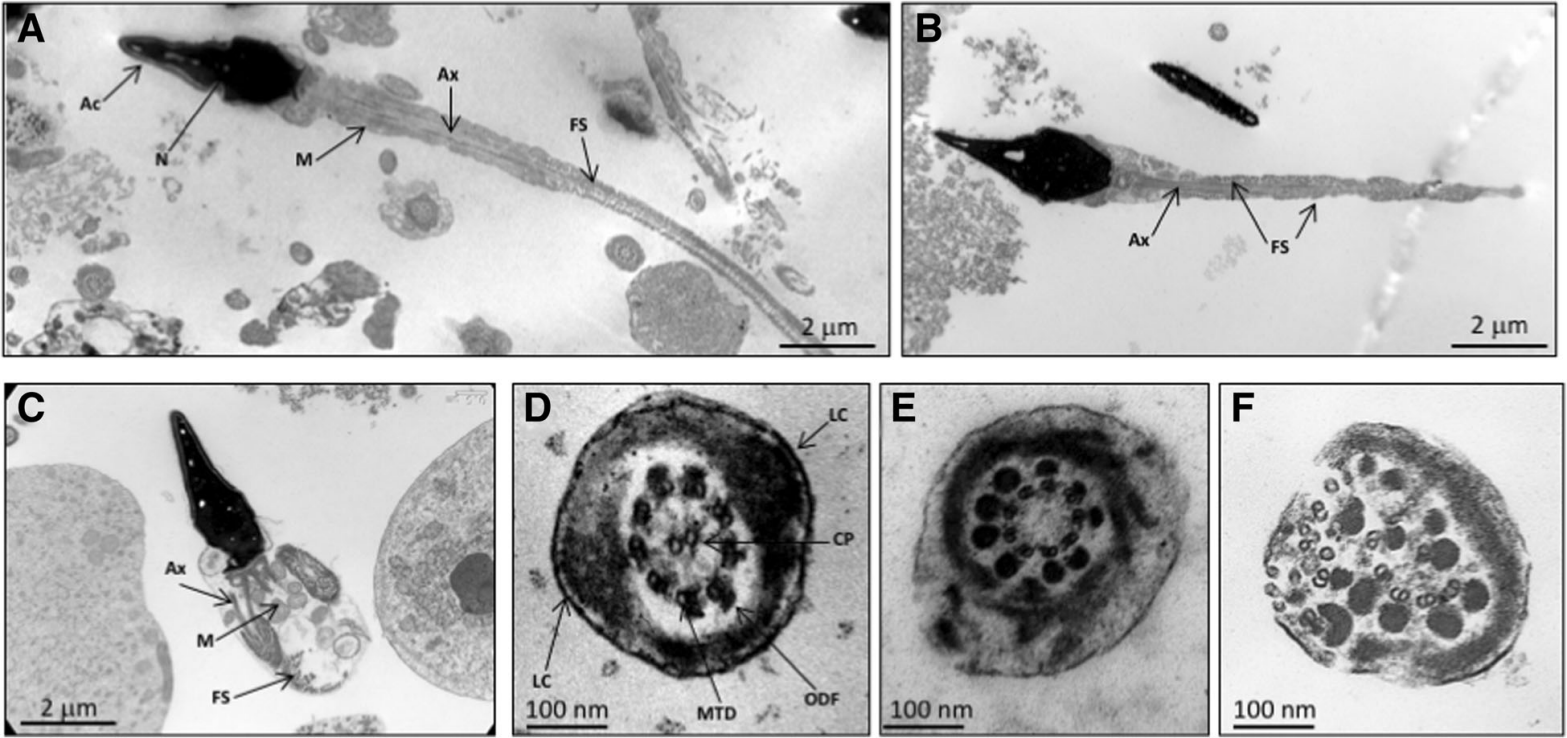
Ultrastructural defects of the MMAF phenotype. **a**, **d** control individual; (**b**, **c**, **e**, **f**) MMAF individual. Pictures from Aminata Touré. **a** Human spermatozoa with the head on the left, and the flagellum on the right side. The flagellum is divided into two main compartments: the midpiece, which comprises the mitochondrial sheath, and the principal piece, characterized by the presence of a fibrous sheath surrounding the axoneme. **b**, **c** Sperm from MMAF individual display incomplete flagellum with short midpiece and abnormal fibrous sheath disposition (**b**); some sperm lack flagellum and display a large cytoplasmic bag with unassembled axonemal and peri-axonemal components (**c**). **d** Transversal section of the axoneme showing the regular microtubule organization with 9 microtubule doublets surrounding the central pair (9 + 2), in normal sperm. **e**, **f** In MMAF individual, the axoneme often display a lack of the central pair or total disorganization. *Ac: acrosome; Ax: axoneme; CP: central pair, ODF: outer dense fibers; FS: fibrous sheath; LC: longitudinal column, MTD: microtubule doublets, M: mitochondria; N: nucleus*

At the structural level, analyses of sperm from MMAF patients show severe defects of flagellum assembly and organization, which contrast with the regular microtubule-based structure of the flagella observed in control sperm (Fig. 2). In particular, the presence of large cytoplasmic bags with unassembled microtubule elements are often observed in due place of the flagella. In addition, in the few sperm bearing a flagellum, the normal microtubule-based conformation of the axoneme (9 + 2) is not apparent but found disorganized with an absence of the central pair and peripheral doublets. The longitudinal columns and fibrous sheath, which constitute the peri-axonemal structures, are also abnormal.

In humans, due to the conserved axonemal structure of motile cilia and sperm flagella, structural and/or functional defects of these organelles are known to cause Primary Ciliary Dyskinesia (PCD). PCD is characterized by recurrent respiratory tract infections, chronic otitis media; in half cases, patients display situs inversus and most men are also infertile [8]. The sperm phenotypes of PCD patients are poorly characterized, and to date, only one PCD gene (*CCDC39*) was described to induce a MMAF-like phenotype [9]. In this short review, we will describe genes, which were demonstrated to induce isolated male infertility due to MMAF phenotype, in patients with no clinical features of PCD. All identified gene mutations we described below were found to segregate with an autosomal recessive mode of inheritance.

### Identification of MMAF-related genes in a cohort of 78 patients from north African, sub Saharan and Caucasian regions

Morphological and ultra-structural defects observed in the MMAF phenotype were exhaustively documented [3–5] but few genetic investigations were performed until very recently. The first genetic study reported a partial genomic deletion of ***AKAP3*** and ***AKAP4*** genes, which encode for the most abundant proteins of the fibrous sheath [10]. Although *Akap4* gene invalidation in the mouse was shown to induce a MMAF-like phenotype [11], this genomic deletion was identified in a single MMAF infertile man and limited analysis was performed to confirm the pathogenicity of the mutation; this result therefore needs to be confirmed in other patients. Nearly one decade after, Ben Khelifa et al. performed a homozygosity mapping study on a cohort of 20 MMAF consanguineous patients from North Africa. This pioneer work led to the identification of homozygous truncating mutations in ***DNAH1***, which encodes an Inner Arm Heavy Chain Dynein (DNAH1), preferentially expressed in the testis [12, 13]. Lack of the DNAH1 protein in MMAF sperm was associated with global axonemal disorganization including mislocalisation of the peripheral microtubule doublets, absence of the central pair and of the inner dynein arms. Subsequent establishment and analysis of a larger cohort of 78 North African, Sub Saharan and Caucasian individuals by exome sequencing, confirmed *DNAH1* as a MMAF gene, accounting for 7.69% of the cases in those populations [6]. Surprisingly, in mouse deletion of DNAH1 induces asthenozoospermia and reduced ciliary beating [14] but in humans no PCD clinical manifestations were reported in the MMAF patients [12], suggesting differences in species or mutation types.

Following this initial discovery, five additional genes were identified by further characterization of the same cohort of 78 MMAF individuals: *CFAP43*, *CFAP44*, *CFAP69*, *FSIP2*, *WDR66/CFAP251* [6, 15–17] .

Mutations in *CFAP43* and *CFAP44*, encoding for WD repeat domains (WDR) containing proteins were identified in 16 patients of the cohort and account for 12.8 and 7.7%, respectively of the MMAF cases. Importantly, invalidation of the orthologous genes in the mouse resulted in male infertility, total sperm immotility and axonemal defects, although a MMAF phenotype was only observed for *Cfap43*^−/−^ mice [6]. RNAi-derived cell lines of the orthologous genes in the flagellated parasite *T. brucei*, displayed growth defects, abnormal flagellum beating, axonemal disorganization but normal flagellum length [6]. The function and precise localization of CFPA43 and CFAP44 proteins in human sperm are unknown; however, recent work in Tetrahymena and Chlamydomonas reported localization of both proteins in the (T/TH) complex, which connects dynein motor domains to ciliary microtubules doublets [18–20]. In *T. brucei,* which harbours a peri-axonemal structure called the paraflagellar rod, the orthologous proteins TbCFAP43 and TbCFAP44 are not uniformly distributed within the axoneme but primarily observed between the peripheral doublets (5 and 6) and the paraflagellar rod, suggesting a potential role in connecting axonemal and peri-axonemal structures [6]; an additional location in the (T/TH) complex of *T. brucei* need to be investigated.

Mutations in the ***CFAP69*** gene, were identified in two unrelated patients of the cohort, accounting for 2.6% of MMAF cases [17]. CFAP69 protein containing Armadillo-like helical repeats localizes to the midpiece of the sperm flagellum and in the mouse, *Cfap69* gene invalidation was found to recapitulate the MMAF phenotype [17], confirming its implication in the processes of sperm flagellum structure and/or assembly. CFPA69 orthologous protein was found enriched in flagellar fraction of Chlamydomonas [21, 22] and interestingly, it is also enriched in cilia from mouse olfactory sensory neurons where it regulates the odour-response kinetics but has no apparent function in structure nor organization [23]. While, based on the mouse model phenotype, no ciliary structural defects of the olfactory neurons would be expected in *CFAP69* mutated patients, further investigations of these patients should be performed to clearly rule out any olfactory functional symptoms.

Mutations in ***FSIP2***, encoding for the Fibrous Sheath (FS) Integration Protein were identified in four unrelated patients, accounting for 5.1% in the cohort [15]. These mutations were associated with a complete disorganization of the FS and axonemal defects. In addition, the absence of AKAP4 protein, known to interact with FSIP2 [24], was observed in sperm from *FSIP2* mutated patients, a feature not observed in patients carrying mutations in other MMAF genes.

Genomic deletion in ***WDR66*** (also named *CFAP251*), encoding for another WDR-containing protein, was identified in 7 patients, which accounts for nearly 9% of MMAF cases of the cohort described by Ben Khelifa et al. [16]. Interestingly, the deletion affects the carboxy-terminal region of *WDR66*, which contains a calcium regulating EF-hand domain. The pathogenicity of the *WDR66* genomic deletion was confirmed by mutagenesis in *T. brucei*, as deletion of the same region in Tb*WDR66*, impaired flagellar structure and movement of the parasite [16]. Although WDR66 function in humans remains to be determined, it was shown to locate to the sperm flagellum in humans. In addition, in the unicellular flagellated alga *Chlamydomonas*, the CFAP251 protein locates to the radial spokes, which connect the peripheral doublets to the central pair of the axoneme [25] and in the ciliated protozoa Tetrahymena, CFAP251 is required for efficient waveform and coordinated ciliary beating [26].

Lastly, a homozygous missense mutation in ***AK7***, a gene encoding for an adenylate kinase expressed in both cilia and sperm flagellum, was identified in two MMAF siblings [27]. Several adenylate kinases were reported as components of axonemal and peri-axonemal structures in mouse sperm flagella (AK1, AK2, AK7 and AK8) [28, 29] and in flagellated protists such as *T. brucei* (ADK1 and ADKB, ADKE) [30] and Chlamydomonas [31]. In mammals, among AK family members, AK7 is the only one harbouring a DPY30 domain, known to be involved in the interaction with AKAP proteins [32]; this feature may contribute to a specific targeting of AK activity close to axonemal components, such as dynein. While no PCD clinical features were observed in the siblings carrying AK7 mutation, the invalidation of AK7 in the mouse induces a severe PCD phenotype including hydrocephalus, respiratory and ciliary defects together with impaired spermatogenesis [33].

### MMAF-related gene mutations in other ethnical populations

Importantly, nearly all above identified MMAF-related genes were also reported to be mutated in patients from different ethnical origins, strongly confirming their pathogenicity. In particular, several studies identified gene mutations in the Chinese population. Wang et al. identified a unique truncating mutation in *DNAH1* in four out of nine MMAF individuals, which they found to only affect the East Asian group. Sha et al. also studied 21 MMAF patients of Han ethnicity and identified DNAH1 mutations in more than half of the patients [34, 35]. In addition, DNAH1 mutations were identified in Iranian and Italian MMAF individuals [36]. The CFAP43, CFAP44, *CFAP69* and CFPA251 genes were also incriminated in the Chinese population [37–39]. In particular, the analysis of 30 MMAF individuals performed by Tang et al. identified mutations in *CFAP43* and *CFAP44* as major cause of MMAF-related infertility in Han Chinese population, with a frequency of 10 and 3%, respectively [38]. Lastly, Auguste et al. identified homozygous truncating mutations in the *WDR66* gene in two MMAF siblings from Lebanon and a third unrelated MMAF individual, further confirming *WDR66* implication in the MMAF phenotype [40].

### MMAF-related gene mutations and PCD phenotype

Although preferentially expressed in the testis and the sperm cells, some of the above MMAF-related genes are detected at low expression level in ciliated tissues and in particular in the lung (see public expression databases); in addition, their encoded proteins are occasionally found in proteomic analyses performed on respiratory ciliated cells [41]. MMAF patients included in the above studies do not display any obvious PCD symptoms but it may be difficult to ascertain the phenotype of isolated MMAF and exclude a weak PCD phenotype, when no functional and structural analyses can be performed on respiratory ciliated cells from the MMAF patients. Despite some limits due to species differences, the use of KO mouse models is possible to answer this point; for instance, ciliary defects and PCD-like phenotype potentially associated with CFAP43 and CFPA44 mutations were excluded based on KO mouse models analyses (Coutton et al. unpublished data). However in some cases, it still remains difficult to have a clear-cut answer, as illustrated for DNAH1, which deletion induces a PCD and male infertility phenotype in the mouse [14] while truncating mutations in humans undoubtedly cause a MMAF phenotype (8% frequency) and only a unique rare missense mutation was so far reported to segregate with a PCD phenotype in humans [42]. An additional level of complexity results from the type of mutation, which may differently impact cilia and sperm cells and cause different phenotypes in those organelles. This point is documented with AK7 gene, which loss of function in the mouse induces a PCD phenotype with male infertility and MMAF phenotype [27, 33], while a missense mutation identified in two MMAF brothers, induces the absence of AK7 protein and axonemal defects in the sperm but no protein damage, nor cilia structure and function in respiratory cells from the patient [27].

Overall, this suggests a possible phenotypic continuum ranging from infertile PCD patients to MMAF patients with no or subtle PCD symptoms; it also emphasizes the complexity in identifying gene mutations strictly involved in a MMAF phenotype, when those genes encode for axonemal components present in both cilia and flagella.

## Conclusion

In the couple last years, genetic studies succeeded in formally identifying 7 novel genes whose mutations account 45% of the 78 MMAF subjects analysed (Table 1). Such successful outcome relies on the efficient combination of high-throughput sequencing technologies together with robust and complementary approaches for functional validation, in vitro, and in vivo using the mouse and unicellular organism models such as the flagellated parasite *T. brucei*.

**Table 1:** Summary table of genes identified in patients displaying a MMAF phenotype

				Mutant models			
Gene	Mutation Frequency	Protein features	Protein localisation	Mouse model	Chlamydomonas	Tetrahymena	Trypanosoma
*DNAH1*	7.69%	Dynein Heavy chain	- Inner Dynein Arm [14]	*MDHC7* mutant: Reduced ciliary beating and asthenozoospermia (no MMAF-like phenotype) [14]	*dhc1b/dhc2* mutant: short flagella [13]	-	-
*CFAP43*	12.8%	WD repeat domains	- cilia associated and component of the (T/TH) complex connected to inner dynein arm [18–20] (Tetrahymena, Chlamydomonas), - flagellar protein located between doublet microtubules 5 and 6 and paraflagellar rod [6] (Trypanosoma)	Asthenozoospermia (MMAF-like phenotype) [6]	–	*Fap43* gene deletion: Altered waveform, beat stroke and reduced swimming speed [18]	*TbCfap43* (Tb.927.4.5380) RNAi mutant: Cell growth defects, Abnormal flagellum beating (axonemal disorganization), normal flagellum length [6]
*CFAP44*	7.7%	WD repeat domains	- cilia associated and component of the (T/TH) complex connected to inner dynein arm [18–20] (Tetrahymena, Chlamydomonas), - flagellar protein located between doublet microtubules 5 and 6 and paraflagellar rod [6] (Trypanosoma)	Asthenozoospermia (no MMAF like phenotype) [6]	–	*Fap44* gene deletion: Altered waveform, beat stroke and reduced swimming speed [18]	*TbCfap44* (Tb.927.7.3560) RNAi mutant: Cell growth defects, Abnormal flagellum beating (axonemal disorganization), normal flagellum length [6]
*CFAP69*	2.6%	Armadillo-type α-helical repeats	- cilia from olfactory sensory neurons [23], sperm flagellum midpiece (mammals) [17], - flagellar associated protein (Chlamydomonas) [21, 22]	Olfactory defects [23] and Asthenozoospermia (MMAF-like phenotype) [17]	–	–	–
*FSIP2*	5.1%	AKAP4 Interacting domain	- sperm flagellum fibrous sheath (mammals) [24]	–	–	–	–
*WDR66 (CFAP251)*	9%	calcium regulating EF-hand domain	- sperm flagellum (mammals) [16], - flagella (Trypanosoma) [16], - radial spoke component (Chlamydomonas, Tetrahymena) [25, 26],	–	–	*Fap251* mutant: Reduced cell swimming, Impaired ciliary beating coordination, normal length [26]	*Tbwdr66* (Tb927.3.1670) deletion: Abnormal motility (axonemal disorganization), growth defects, normal flagellum length [16]
*AK7*	1.28%	ADK domain, coiled-coil domain, DPY30 domain	- cilia and sperm flagella (mammals) [27, 33].	PCD [33] and Asthenozoospermia (MMAF-like phenotype) [27]	–	–	–

This work clearly contributes to improving the genetic diagnosis provided to patients; further studies should provide genotype-phenotype correlations, which could be helpful for the prognosis of intracytoplasmic sperm injection (ICSI) performed with sperm from MMAF patients. Altogether, this should ameliorate the clinical care provided to couples in the course of Assisted Reproduction Technologies. In addition, this work also improves our knowledge about the molecular mechanisms involved in sperm flagellum structure and beating. In this regard, data provided by ultra-structural, proteomic and mutagenesis studies performed in unicellular organisms, such as Trypanosoma, Tetrahymena and Chlamydomonas (Table 1) provide important clues to the functions and axonemal localisation of the proteins encoded by these identified MMAF.

Although, the functions and molecular mechanisms of action of most proteins encoded by the MMAF-genes in humans are still unknown, part of them contain protein motifs known to be associated with scaffold protein properties. These features together with their location to multiple subcellular compartments of the sperm flagellum, ranging from the axoneme (DNAH1, WDR66), the peri-axonemal structures (AKAP, FSIP2), the axonemo-periaxonemal space (CFAP43, CFAP44) and the midpiece (CFAP69), obviously emphasize the complexity of the protein networks and molecular mechanisms, which are likely to govern sperm flagellum assembly, organization and beating.

## Abbreviations

AK: Adenylate Kinase
AKAP: A-kinase Anchoring protein
CFAP: Cilia and Flagella Associated Protein
FSIP2: Fibrous Sheath Interacting Protein
MMAF: Multiple Morphological Abnormalities of the sperm Flagellum
PCD: Primary Ciliary Dyskinesia
WDR: WD Repeat domain
